# Capturing Usability Problems for People Living With Dementia by Applying the DEMIGNED Principles in Usability Evaluation Methods: Mixed Methods Study

**DOI:** 10.2196/54032

**Published:** 2024-07-31

**Authors:** Thomas Engelsma, Simone Heijmink, Heleen M A Hendriksen, Leonie N C Visser, Afina W Lemstra, Monique W M Jaspers, Linda W P Peute

**Affiliations:** 1 eHealth Living & Learning Lab Amsterdam Department of Medical Informatics Amsterdam UMC, location University of Amsterdam Amsterdam Netherlands; 2 Amsterdam Public Health Digital Health Amsterdam Netherlands; 3 Amsterdam Public Health Aging & Later Life Amsterdam Netherlands; 4 Alzheimer Center Amsterdam Neurology, Vrije Universiteit Amsterdam Amsterdam UMC, Location VUmc Amsterdam Netherlands; 5 Amsterdam Neuroscience Neurodegeneration Amsterdam Netherlands; 6 Amsterdam Public Health Quality of Care Amsterdam Netherlands; 7 Division of Clinical Geriatrics, Center for Alzheimer Research Department of Neurobiology, Care Sciences and Society Karolinska Institutet Stockholm Sweden; 8 Department of Medical Psychology Amsterdam UMC location University of Amsterdam Amsterdam Netherlands

**Keywords:** dementia, design principles, digital health, memory clinic, usability evaluation, mobile phone

## Abstract

**Background:**

Dementia-related impairments can cause complex barriers to access, use, and adopt digital health technologies (DHTs). These barriers can contribute to digital health inequities. Therefore, literature-based design principles called DEMIGNED have been developed to support the design and evaluation of DHTs for this rapidly increasing population.

**Objective:**

This study aims to apply the DEMIGNED principles in usability evaluation methods to (1) capture usability problems on a mobile website providing information resources for people visiting a memory clinic, including those living with subjective cognitive decline (SCD), mild cognitive impairment (MCI), or dementia, and (2) investigate the realness of usability problems captured by the DEMIGNED principles in expert testing, specifically for mobile websites that act as a means of providing DHTs.

**Methods:**

First, a heuristic evaluation was conducted, with the DEMIGNED principles serving as domain-specific guidelines, with 3 double experts (experienced in both usability and dementia) and 2 usability engineering experts. Second, think-aloud sessions were conducted with patients visiting a memory clinic who were living with SCD, MCI, or dementia.

**Results:**

The heuristic evaluation resulted in 36 unique usability problems. A representative sample of 7 people visiting a memory clinic participated in a think-aloud session, including 4 (57%) with SCD, 1 (14%) with MCI, and 2 (29%) with dementia. The analysis of the think-aloud sessions revealed 181 encounters with usability problems. Of these encounters, 144 (79.6%) could be mapped to 18 usability problems identified in the heuristic evaluation. The remaining 37 (20.4%) encounters from the user testing revealed another 10 unique usability problems. Usability problems frequently described in the think-aloud sessions encompassed difficulties with using the search function, discrepancies between the user’s expectations and the content organization, the need for scrolling, information overload, and unclear system feedback.

**Conclusions:**

By applying the DEMIGNED principles in expert testing, evaluators were able to capture 79.6% (144/181) of all usability problem encounters in the user testing of a mobile website for people visiting a memory clinic, including people living with dementia. Regarding unique usability problems, 50% (18/36) of the unique usability problems identified during the heuristic evaluation were captured by the user-testing sessions. Future research should look into the applicability of the DEMIGNED principles to other digital health functionalities to increase the accessibility of digital health and decrease digital health inequity for this complex and rapidly increasing population.

## Introduction

### Background

Digital health technologies (DHTs) have the potential to improve health outcomes, information access, patient monitoring and self-management, treatment adherence, and disease diagnostics and prevention [1-4]. However, susceptible patient groups, such as older adults or people with disabilities, can experience challenges with accessing DHTs, creating digital health inequities [5,6]. Besides well-known accessibility issues such as financial or geographical barriers, human factors such as insufficient digital skills, poor motivation, low health literacy, poor health conditions, low socioeconomic status, and declining cognitive and physical abilities can also decrease the accessibility of DHTs [5,7-9]. These factors may also result in specific challenges to using DHTs that should be accounted for during the development of DHTs to increase their usability. Usability is defined as the extent to which DHTs can be used to achieve specified goals with effectiveness, efficiency, and satisfaction [10]. Problems related to the usability of DHTs arise when their design is not tailored to the needs of specific end users, thereby hampering their acceptability, user engagement, adoption, and successful use [2,11-13].

A vulnerable patient group that can experience challenges with accessing and using DHTs is people living with dementia. The prevalence of dementia is increasing globally as the population ages, making it a significant public health concern. It is expected that >150 million people in 2050 will be living with dementia, with 10 million new cases each year [14]. There are many underlying causes of dementia. Therefore, different types of dementia can be distinguished, such as dementia due to Alzheimer disease, vascular dementia, Lewy body dementia, and frontotemporal dementia [15]. People living with dementia face specific barriers when trying to use DHTs, which can worsen disparities in digital health care access [9,16]. These barriers relate to their cognitive, perceptive, and physical decline; changing frame of mind; and decreasing speech and language skills [9]. Even though these barriers to using DHTs for people living with dementia exist, the availability of DHTs increases, as they are suggested to improve the quality of life for this population by providing, among others, assistance with activities of daily living; improve their social engagement; and monitor their cognitive functions [17-19].

### Objectives

To improve the usability and accessibility of DHTs for people living with dementia, their design can benefit from context-specific design guidance. While accessibility standards presented in, for example, International Organization for Standardization Technical Specification 82304-2 [20], the Web Content Accessibility Guidelines [21], and xCertia Guidelines [22] can serve as valuable foundations for inclusive design, “understanding user diversity and applying this in the development process” [23], their use during the development of DHTs and evaluation is currently limited [24]. In addition, these standards and guidelines may not comprehensively address the complex, unique, and multifaceted needs of vulnerable populations, such as people living with dementia or other cognitive and physical impairments [24-26]. Ensuring the accessibility and usability of DHTs for such vulnerable user groups necessitates the implementation of additional context-related and detailed design specifications, something often missing in traditional guidelines and principles [24]. Therefore, in previous research, we developed literature-based design principles, or DEMIGNED principles, to be considered when developing DHTs for this population [27,28]. However, empirical evidence needs to be collected to further investigate the applicability of the DEMIGNED principles and their potential refinements. To collect this empirical evidence, this study aimed to apply the DEMIGNED principles in usability evaluation methods to (1) capture usability problems on a mobile website providing information resources for people visiting a memory clinic, including those living with subjective cognitive complaints, mild cognitive impairment (MCI), or dementia, and (2) investigate the realness of usability problems captured by the DEMIGNED principles in expert testing, specifically for mobile websites that act as a means of providing DHTs.

## Methods

### Study Design

Investigating the realness of usability problems captured by the DEMIGNED principles can encompass usability evaluation research [29]. According to Hartson et al [30], a usability problem is real if it is “a predictor of a problem that users will encounter in real work-context usage and that will have an impact on usability (user performance, productivity, and/or satisfaction)” [30]. Therefore, in the first part of this study, usability issues on a mobile website for patients visiting a memory clinic, including people with dementia, were captured by applying the DEMIGNED principles. This was conducted through a heuristic evaluation approach. A heuristic evaluation is a low-cost method where usability or domain experts evaluate a system’s navigation structure, screen layout, and interaction structure, typically on a set of predefined generic heuristics or guidelines [31]. This resulted in a list of unique usability problems that violate a specific heuristic or guideline. To investigate the realness of these usability problems, these findings were used to map the results from inclusive user-based testing with patients visiting a memory clinic with subjective cognitive decline (SCD), MCI, or dementia. As there are challenges to inclusive-based user testing with people living with dementia, we applied considerations proposed by human factors experts to build trust and decrease potential stress [32]. To conduct the 2 usability evaluations, the mobile website of Alzheimer Center Amsterdam, a diagnostic and treatment center for (early-onset) dementia, was examined [33]. Their website contains information and other resources for people who are diagnosed with (or concerned about having) dementia and their relatives, researchers, funders, and others interested in learning about dementia (research). On the basis of the findings, recommendations for redesign are presented.

### Heuristic Evaluation Approach

By applying the heuristic evaluation method, the overall user interface and structure of the mobile website of Alzheimer Center Amsterdam were assessed. In this study, potential usability problems were captured by assessing violations of the DEMIGNED principles (Table 1). These principles were developed by TE, LWPP, and MWMJ [27,28]. The design principles have been mapped to the categories of the mobile health for older adults with dementia–usability framework (MOLDEM-US), which captures barriers to using DHTs, specifically mobile health technologies, for people living with dementia [9]. These categories of barriers relate to cognition, perception, frame of mind, and speech and language.

**Table 1:** Overview of the DEMIGNED design principles for digital health technologies for people living with dementia.

Category and design principle		Abbreviation^a^
**Cognition**		
	Support the monitoring of action progress	C-monitoring
	Provide tutorials with short instructions to guide the digital tool	C-tutorials
	Provide functionalities and actions adjustable to the user’s cognitive abilities	C-abilities
	Allow easy navigation to functions and content in a digital tool	C-navigation
	Implement representative and understandable icons	C-icon use
**Perception**		
	Provide visually compartmentalized user interfaces	P-compartmentalize
	Provide appropriate system feedback	P-system feedback
	Implement distinguishable colors	P-color use
	Allow distinguishable clickable and nonclickable areas	P-click ability
	Allow easily processable elements	P-elements
**Frame of mind**		
	Provide continuous support	F-support
	Ensure no time pressure	F-time
	Provide positive feedback for correct action completion	F-positive feedback
	Implement app settings adjustable to personal preferences	F-preferences
	Provide attractive and respectful content	F-content
**Speech and language**		
	Ensure the use of understandable words and sentences that feel comfortable	S-understandability
	Allow user input through both speech and text	S-user input

^a^Each principle has an abbreviation presented with the first letter of the category, followed by a keyword describing the principle.

### Participants and Procedures

Previous heuristic evaluations have shown that 3 to 5 experts can identify 74% to 87% of usability problems [34]. This evaluation was performed by 5 evaluators: 3 double experts, TE, SH, and LWPP (knowledgeable in both usability engineering and dementia), and 2 usability engineering experts (Table 2). After confirming participation, TE familiarized the evaluators with the DEMIGNED principles in an introductory session where the evaluators were able to discuss and ask for clarifications on the principles.

A total of 3 use cases were determined based on the information presented on the website: patient care, scientific research, and information about dementia. The evaluators assessed these use cases in an overall manner to gain a sense of the website’s navigation and structure before a more thorough assessment of the user interface components. Because of the high number of pages on the website and the uncertainty of which pages participants will encounter in the think-aloud session, example tasks were composed for each use case to guide the evaluation (Table 3).

The DEMIGNED principles were used as a set of heuristics by the evaluators and applied to check the design of the website’s user interface and structure. If a principle was violated, it was given a severity rating based on the following scales, defined by Nielsen [35]: “(0) I do not agree that this is a usability problem at all (1) Cosmetic problem only: need not to be fixed unless extra time is available on project, (2) Minor usability problem: fixing this should be given low priority, (3) Major usability problem: important to fix, so should be given high priority or (4) Usability catastrophe: imperative to fix this before the product can be released.” Moreover, the evaluators reported for each identified usability issue the location on the website and the violated DEMIGNED principle. Usability issues can be related to multiple DEMIGNED principles, allowing the experts to report >1 principle for a unique usability issue.

**Table 2:** Overview of experts participating in the heuristic evaluation.

Degree	Sex	Research expertise	Experience (years), n	Occupation
PhD	Female	Human factors engineering in health care	20	Senior researcher
MSc	Female	User experience	8	UX^a^ designer and university lecturer
MSc	Male	Design for people living with dementia	6	Assistant professor
MSc	Female	Design for vulnerable populations	1	PhD student
BSc	Female	Working with people living with dementia and experience with HE^b^	3	Medical informatics student

^a^UX: user experience.

^b^HE: heuristic evaluation.

**Table 3:** Tasks conducted in the heuristic evaluation.

Use case	Task
Patient care	1. Find information about the *Screeningsdag* 2. Find the information video on the lumbar puncture 3. Go to the log-in page of *MijnDossier* 4. Find information on how to participate in scientific research 5. Find the clinician who will see you during the *Screeningsdag* 6. Find the physical address of the center 7. Find the phone number of the center 8. Find information about getting a second opinion
Scientific research	9. Find information about ongoing research projects from the center 10. Find information about completed research projects from the center 11. Find information about hersenonderzoek.nl
Information about dementia	12. Find information about whether you have dementia or not 13. Find information about the treatment of dementia 14. Find tips about how to live with dementia 15. Find personal stories from patients 16. Find the frequent asked questions 17. Find information about getting dementia at a young age 18. Find information about “corticobasal degeneration”

### Data Analysis

The identified usability problems were coded by performing a deductive thematic analysis. The DEMIGNED principles were used to predefine usability themes in which issues could arise. All usability issue encounters were combined in 1 master list by SH, after which duplicates were summed independently by TE and SH. The issues were summed overall rather than per task, as the aim was to obtain an overall report of the usability issues the DEMIGNED principles can capture on a mobile website. This led to a final set of unique usability issues on which consensus was reached with LWPP. The severity ranking for each unique issue was calculated by taking the average severity given by the evaluators who identified the usability issue. It was decided to report an average severity score rather than a consensus score to moderate potential extreme views in assessing usability problems, given the novelty of the DEMIGNED principles. This offers a balanced representation of the severity score, eliminating the need for evaluators to reach a consensus.

### Think-Aloud Method

#### Participants

For the think-aloud sessions, participants were recruited at Alzheimer Center Amsterdam. The aim of the recruitment was to include a representative sample of the (heterogenous) population that is most likely to use the website of Alzheimer Center Amsterdam: those who visit the memory clinic to seek support for their cognitive complaints. Alzheimer Center Amsterdam has a focus on patients living with dementia at a younger age (<65 years). Within this group, the most frequent diagnosis is SCD, followed by dementia and MCI [36]. Therefore, for the sample to be representative, more people with SCD were recruited, followed by people with dementia and MCI. All participants were presented as patients at the memory clinic of Alzheimer Center Amsterdam, where they received a standardized dementia diagnostic workup. Clinical diagnosis was made in a multidisciplinary meeting and discussed with patients during a second appointment. Subsequently, patients were offered annual or biannual follow-up. People scheduled for such a follow-up appointment were called to participate when they had previously given permission to be approached for research; had an appointment on June 5, June 8, or July 6, 2023; were not already participating in other research (that day); and were Dutch speaking. Patients who were unable to give informed consent were excluded from participation. Those who showed interest in participating received an information letter. In addition, a follow-up telephone call was scheduled to answer any questions and confirm participation after participants had the opportunity to read the information letter in depth.

#### Study Procedures

After confirming participation, a think-aloud session was scheduled either before or after the participant’s appointment at Alzheimer Center Amsterdam. We aimed to adopt an empathetic approach to ensure there was trust between the participant and the evaluators (LWPP, SH, and TE) and to make the participant feel comfortable [32]. After a short informal chat, participants provided informed consent and completed a paper-based questionnaire about their age, sex, and technology use. Thereafter, the think-aloud session started. The participant was then asked to sit behind the smartphone. The researcher sitting next to the participant first explained the goal of the session and showed how to verbalize thoughts through an example task on the smartphone provided by the researcher (eg, “Find what the weather will be this Saturday.”). Participants were then asked to complete 7 tasks on the mobile website while verbalizing their thoughts (Textbox 1). For each use case, these tasks were derived from the example tasks for the heuristic evaluation but were specified to specific end points deemed relevant to the end users, such as information resources. The completion of the tasks was monitored by a second researcher, sitting behind the computer that recorded the session and across from the participant. If a partner or relative was present, they were instructed to only provide motivational reactions if the participant became silent. Therefore, only usability problems detected by the participants were gathered and analyzed. Even though this may introduce bias, this decision was made to make the participant feel more comfortable and simulate a more real-life setting for the participant [32].

The facilities at eHealth Living & Learning Lab Amsterdam were used to both audio record and video record the think-aloud session. This allowed researchers to capture rich data from both the user’s interactions with the mobile device and the verbalizations from the think-aloud session. The facilities used to capture these data include (1) a video camera to record the participant’s hand interaction with the smartphone, (2) a voice recorder to record the verbalized thoughts of the participant, (3) a smartphone device for the participant to access the mobile website, and (4) two laptops. One laptop was used to capture the screen from the smartphone through screen casting. The other laptop was used for Viso software (Noldus) to bring together the video recording, the screen capture recording, and the audio recording [37].

**The 7 tasks conducted in the think-aloud sessions.**

**Tasks**

1. Find the date of the next event of Alzheimer Center Amsterdam
2. Find the 4 health care professionals scheduled for the screening day
3. Find information about the nurse consultants
4. Find the video about lumbar puncture
5. Find the conversation guide for the screening day
6. Find information about hersenonderzoek.nl
7. Find details about a fitness to drive statement

#### Data Analysis

First, all audio recordings were transcribed. Segments that did not convey verbal thoughts, such as instances when the participants read the website, were excluded. Second, the video recordings were analyzed and used to amplify the transcribed audio recordings. Third, the resulting transcripts were openly coded first and axially encoded afterward. Finally, the frequency of problems per category was registered. The resulting usability problems were mapped onto the findings from the heuristic evaluation by TE, SH, and LWPP. Each usability problem encounter was mapped onto a theme from the DEMIGNED principles (cognition, perception, frame of mind, or speech and language). Afterward, if applicable, a subtheme from the heuristic evaluation findings was linked to the usability problem encounter, followed by a specific issue. Findings from the think-aloud sessions that could not be verified with the findings from the heuristic evaluation will be presented separately.

### Ethical Considerations

The study was approved by the Amsterdam University Medical Center medical ethical review committee with the number 2023.0240. All participants received detailed information about the study and provided written informed consent before participation in the think-aloud study. All data used in analysis have been anonymized. Each participant in the think aloud sessions received a €10 (US $10.90) gift card.

## Results

### Heuristic Evaluation Approach

A total of 4 evaluators completed the heuristic evaluation. One evaluator conducted only the example tasks related to patient care and was further involved in the development of the master list. The heuristic evaluation resulted in a final set of 36 unique usability issues, as shown in Multimedia Appendix 1. The usability issues identified in the cognition theme can impact people living with dementia when interacting with the website. The malfunctioning search function, nonintuitive navigation processes, misaligned information headings, inconsistent structures, nonlinear pathways, duplicated content, and scrolling difficulties can lead to confusion and fatigue. In addition, the lack of logical menu structures, faulty filter functions, and challenges in finding essential features such as the information videos and the patient portal can exacerbate cognitive impairments associated with dementia, hindering effective navigation and information retrieval. Furthermore, external navigation links may disrupt the user’s cognitive flow and understanding.

### Think-Aloud Method

A total of 7 participants were included in the think-aloud sessions, composing a representative sample of people visiting the memory clinic at Alzheimer Center Amsterdam (Table 4). Each session lasted approximately 30 to 50 minutes. A total of 5 audio recordings and 7 video recordings were used in the analysis.

The think-aloud sessions revealed 181 usability problem encounters, of which 144 (79.6%) were mapped to 18 usability problems identified during expert testing (Multimedia Appendix 1). Most frequent usability problem encounters that verified the findings from the heuristic evaluation relate to the user expectations (48/181, 26.5%), information overload (20/181, 11%), system feedback (19/181, 10.5%), search function results (12/181, 6.6%), and scrolling (9/181, 5%). Examples of these issues are provided by means of quotes in Table 5.

**Table 4:** Characteristics of patients visiting a memory clinic who participated in the think-aloud sessions (n=7).

Characteristics		Values, n (%)
**Diagnosis**		
	Subjective cognitive decline	4 (57)
	Mild cognitive impairment	1 (14)
	Dementia	2 (29)
**Sex**		
	Male	3 (43)
	Female	4 (57)
**Age (years)**		
	50-59	2 (29)
	60-69	2 (29)
	70-79	2 (29)
	80-89	1 (14)
**Smartphone use**		
	Yes	6 (86)
	No	1 (14)
**Smartphone use (min/day)**		
	0-30	2 (29)
	30-60	2 (29)
	60-120	1 (14)
	>120	1 (14)
**Tablet use**		
	Yes	5 (71)
	No	2 (29)
**Tablet use (min/day)**		
	0-30	3 (43)
	30-60	2 (29)
**First time viewing the website**		
	Yes	3 (43)
	No	4 (57)

**Table 5:** Example quotes from transcript analysis for the most frequently encountered usability problems during the think-aloud sessions.

Usability problem	Example quote from transcript analysis
User expectations	“I wouldn’t know how to search for this and under which heading it falls. [Read aloud: about dementia, patient care, professionals, scientific research]. I don't see a section called ‘what if you've received a diagnosis.’ I can't find it; I wouldn't know” (Participant 3). “My Record [Dutch: ‘MijnDossier,’ a patient portal]. When I click on that, there’s nothing about me personally. My record means my record, but there are general things there. And I would like to access my record, and it exists because it’s listed here below, if I see it correctly” (Participant 1).
Information overload	“Well, I just saw it, and I’m just trying to remember where. They’re, of course, trying to provide a lot of information” (Participant 2). “Forms of dementia. [Looks at the menu screen for a while]. Yes, there’s so much on it; it’s making me a bit fidgety, and then I think, what was it I wanted to look up again? I’m getting completely distracted by all those things“ (Participant 5).
System feedback	“What I notice here is that there are long lists, and you’re working with people who have dementia or the caregivers, of course. With those long lists, you touch them, and nothing happens, but then the information is listed below” (Participant 4). “Yes, I was just there earlier. Then we go back to the preparation. [Clicks three times on ‘preparation’ in the menu but doesn’t see anything change]” (Participant 2).
Search function	[Searches for “lumbar puncture” in the search bar; clicks on a result.] “I have the protein research here, so that’s a lumbar puncture. That’s what you’re looking for, right? Or are you looking for what it looks like?” (Participant 3) “Let me see. I’ve entered your question, and I actually expect an answer, but it’s not doing that. Yes, because when I do this on Google, I get 20 answers, and then I can choose. But here, I don’t get there” (Participant 1).
Scrolling	“They naturally have a lot of information, and then you click on this, and it’s listed below [under the menu], and personally, you know, I just want to see it at the top because, yeah. I've had times when I thought ‘I'm not there or something,’ but it’s actually there.” (Participant 2). “Yes, you see, I have to go down (scroll) every time. Lumbar puncture, well, look” (Participant 1).

### Additional Usability Issues Identified Through the Think-Aloud Analysis

A total of 37 usability problem encounters, solely identified in the think-aloud analysis, were thematically categorized into 10 unique usability problems and further classified into 7 themes (Table 6).

The findings revealed additional issues with identifying and using the search function, such as (recovering from) typing errors; the use of too many search terms leading to an overload of search results; and confusion caused by the visibility of the search history from the mobile device, rather than solely the queries on the website’s search engine. Some participants limited their mental model for information finding solely to the search engine, leading to insecurities when asked to apply other navigation structures, such as clicking through the menu structure. Moreover, irrelevant search results or typing errors caused difficulties with remembering the task they were asked to perform. Finally, 3 additional usability issues related to the participants’ frame of mind while interacting with the website were identified: insecurity, stress, and habit. Participants verbally conveyed questions about their own ability to interact with the website and stressful responses while conducting tasks, and these were coded as shown in Table 6.

**Table 6:** Usability issues identified only through the think-aloud analysis.

Theme and usability problem		Impact	Severity score^a^, average	Frequency of the problem, n
**Touch screen** **sensitivity**				
	The touch screen of the smartphone is too sensitive for participants who experience slower performance speed.	It causes unwanted user actions, such as accidentally pressing external links or pressing too hard instead of swiping or scrolling.	2	2
**Search function issues**				
	Typing errors in a search query when using the search function leads to no or unwanted search results.	When typing error goes unnoticed, the user does not understand why information cannot be found, leading to frustration and inability to complete tasks.	4	6
	The search function cannot be accessed via the home page and can be accessed only after clicking on the menu icon.	If a user limits their mental model for information finding solely to the search function and this cannot be found, it prevents them from completing tasks.	4	3
	Too many search terms in a search query lead to an overload of results.	When the user types “driving and dementia,” many results are shown, including all pages that include “and,” leading to frustration and inability to complete tasks.	4	3
	Search history is shown from when typing a search query.	When a user wants to type a search query in the search bar but the search history appears below the bar, this can be seen as a dropdown menu with an option to select from, causing confusion.	2	1
**Return button**				
	The return button is placed under the menu structure but above the content of the page, making the user miss information.	When a user sees the return button under the menu structure, they expect there is no more content on the page when they do not scroll down. Therefore, they might miss valuable information, preventing them from completing an action.	3	3
**Insecurity**				
	Users verbally question their own interactions with the website while performing tasks.	Insecurity can lead to decreased motivation and willingness to use the website.	2	5
**Stress**				
	Users verbally convey stress while interacting with the website.	Stress can lead to decreased motivation and willingness to use the website.	2	10
**Habit**				
	Users verbalize having their own habits while using websites, which are not aligned with the website under evaluation, such as changing a search query, only using Google search and button recognition.	When a user is too stuck in such habits, it makes it difficult to adjust to the website’s design, causing difficulties in completing a task.	2	3
**Clicks**				
	The user needs too many clicks to find the desired information, while the user expects to find information faster.	The user cannot reach the page to complete a task.	3	1

^a^Average severity score based on Nielsen’s severity ranking [35].

## Discussion

This study aimed to apply the DEMIGNED principles in usability evaluation methods to (1) capture usability problems on a mobile website providing information resources for people visiting a memory clinic, including those living with subjective cognitive complaints, MCI, or dementia, and (2) investigate the realness of usability problems captured by the DEMIGNED principles in expert testing, specifically for mobile websites that act as a means of providing DHTs. In addition, this study provided insights for the future refinements of these principles.

### Principal Findings

The mobile website of Alzheimer Center Amsterdam underwent both heuristic evaluation and evaluation through the think-aloud method to investigate the realness of usability problems detected by applying the DEMIGNED principles in expert testing. To this end, usability problems derived from user testing were mapped to findings from expert testing. The heuristic evaluation, covering most of the website’s pages, revealed 36 distinct usability issues. Subsequently, during the think-aloud sessions with people living with SCD, MCI, or dementia, 18 unique usability problems were mapped to the findings from the heuristic evaluation. In addition, the think-aloud sessions revealed 10 new usability issues. Despite potential differences in the number of pages viewed between the heuristic evaluation and the think-aloud session, given the uncertainty surrounding the information-seeking strategies of participants in the think-aloud session, these results suggest a 50% (18/36) validity score for the DEMIGNED principles in an expert test based on the usability evaluation method validity score measurement [30]. However, this first investigation aimed to explore the realness of usability problems that can be captured by applying the novel DEMIGNED principles, gaining insights into the use of the DEMIGNED principles. Follow-up research should determine the usability evaluation method effectiveness of applying the DEMIGNED principles to detecting usability issues through heuristic testing. Nevertheless, a validity score of 50% (18/36) in this study suggests the potential of the DEMIGNED principles for future heuristic evaluation approaches. This contrasts with other research showing a validity score of 40% when using commonly used heuristics [38]. Moreover, looking at the number of times a usability problem was encountered, 79.6% (144/181) of the usability problem encounters were captured by experts using the DEMIGNED principles.

The findings indicate the potential for the DEMIGNED principles to be further used as a set of guidelines for a heuristic evaluation of DHTs for people living with SCD, MCI, or dementia. This can be valuable, as current sets of heuristics may not sufficiently capture usability issues experienced by people living with dementia. Over time, numerous sets of heuristics have been devised to assess the usability of mobile devices, focusing primarily on their physical attributes and phone interfaces [39,40]. Widely acknowledged heuristics of Nielsen [34] are valuable for evaluating general usability in website interfaces. Nevertheless, these guidelines do not fully encompass the unique challenges associated with designing DHTs for people living with dementia. Heuristics to capture usability problems in the design of DHTs related to cognitive decline, such as memory loss, reduced attention span, and decision-making difficulties, are lacking. Similarly, heuristics proposed by Neto and da Graça Pimentel [41], inspired by Nielsen [34], focus on assessing the usability of mobile apps but lack specific heuristics to capture usability problems experienced by people living with dementia. These heuristics are tailored for general mobile interface design and do not comprehensively address specific barriers to using DHTs for this population, such as attention deficits and challenges in learning and adapting to new interfaces. For medical devices, heuristics proposed by Zhang et al [42] are commonly used. However, these might not be entirely suitable for evaluating DHTs for people living with dementia. Heuristics proposed by Zhang et al [42] prioritize the detection of usability issues for medical devices, such as issues with accuracy, reliability, and safety. However, these are not attuned to capture usability problems caused by, for example, emotional fluctuations or cognitive decline.

### Refinement of the DEMIGNED Principles

The think-aloud sessions revealed 10 usability problems that were not found through expert testing that may require further refinement of the DEMIGNED principles. First, 3 (30%) of these problems relate to the frame of mind of the user. It was observed that interacting with the website can cause verbal reactions of stress and insecurities. This was also the case when a participant had a certain habit of finding web-based information that was not in line with how the evaluated website can be searched. These barriers may lead to unsuccessful task completion and should, therefore, be accounted for and included in the DEMIGNED principles. However, the 3 usability issues related to the frame of mind seemed to be related to navigational structure, suggesting that a more linear navigational structure might make information easier to find and, therefore, reduce stress and insecurity. Second, the design of the search function was revealed to be a critical usability problem that prevented successful task completion. Even though 2 potential usability problems were identified by one of the evaluators during the heuristic evaluation, 3 more issues were revealed in the think-aloud analysis when people with SCD, MCI, or dementia used the engine. Using the search function revealed issues with typing errors, too many search terms leading to an overload of results, and a misunderstanding of the search history. Therefore, the DEMIGNED principle of navigation should provide design guidelines specific to search functions. Finally, 3 usability problems were encountered by a few participants. First, the amount of clicking was found to be too much to find the information they were looking for, which was also found in other studies where participants showed problems finding content because of the navigation structure [43,44]. Limiting the amount of clickable content on a single screen may support directing the users to relevant information [43]. Second, the sensitivity of the touch screen led to unwanted actions, which were caused by the device itself. Currently, the DEMIGNED principles only focus on user interface and structures rather than the hardware; therefore, this problem may not have been found by the evaluators in the heuristic evaluation. However, touch screen sensitivity could be tackled by providing visible or audible system feedback when a user clicks too fast or a pop-up to confirm an action [28]. Third, it was observed that the position of the “return” button above the content caused challenges, as one of the participants mistakenly perceived that no additional information was available below the button. This provides insights into further refining the DEMIGNED principles, such as icon use and system feedback, with guidelines regarding button placement as part of the content organization.

### Recommendations for Redesign

#### Overview

The website of Alzheimer Center Amsterdam not only is informative for people living with SCD, MCI, or dementia and others having an appointment at the memory clinic but also contains information for researchers, funders, family members, etc. However, these recommendations aim to enhance the website’s accessibility and user experience for people living with dementia. Moreover, they can also benefit those interested in redesigning for improved digital health accessibility in this context. Overall, it is suggested to redesign the website with general principles that can improve accessibility, such as by adding an option to read text out loud to the user, increasing the font size without decreasing readability, and magnifying options for images and other graphics. Furthermore, 5 specific areas of redesign were identified based on the findings from this study, namely navigation, information overload, scrolling, content and user experience, and system feedback.

#### Navigation

Navigation often requires sufficient decision-making skills, the awareness of location, and a sequence of clicks. These skills may decline due to memory impairment, decreased learnability, and difficulties with processing information. Therefore, it is recommended to implement linear navigation. This means the user can move only forward or backward through the website and its content. Starting with the home page, it is recommended to redesign this to full-screen navigation, showing clickable main options to navigate to “patient care,” “scientific research,” and “living with dementia.” After selecting one of these main options, a full-screen menu with suboptions to choose from should open. This may tackle the usability problem of users not noticing that the “main option” is also a page with unique information. Additional research prioritizing information needs and formulating clear suboptions may support this redesign, given the perceived discrepancies between the current information headings and accompanying content. In addition, this requires removing potentially irrelevant, outdated, or duplicate information and empty pages to ease the process of scanning and finding information.

Furthermore, participants experienced challenges with using the search function. In general, people often rely on searching as their main strategy [45]. In this study, those who relied solely on the search function experienced difficulties when navigating the website. Familiarity with search engines such as Google may make them more inclined to use the search feature as their default interaction method [43]. Overall, the availability of a function that eases information finding on a website is important for people living with dementia [43,44]. However, some participants experienced difficulties finding the search function, as it was accessible only after clicking on the menu icon, or using the function, as it did not work as expected. This led to confusion, frustration, poor user experience, and hindered task completion during think-aloud sessions. This reduced mental model of finding information may be caused by barriers related to the user’s frame of mind, such as a lack of trust in their abilities or perceived complexity [9]. In addition, a reason for using the search function as the default interaction method may be due to affected language and communication skills. Therefore, it is suggested to redesign the search function to prevent typing errors, too many and irrelevant results, and the presentation of results in an illogical order. Implementing a Google-like search function with positive system feedback can be considered, where suggestions for typing errors are made, an overview of the most relevant results are presented, and selecting for filters that are of relevance to the user is possible. Such a search function could also be relevant for the “team” page to ease the user’s process of finding information about their physician. Moreover, the search function can be placed on the home page to increase its accessibility.

#### Information Overload

An excessive amount of information on a single page and in the menu (eg, the number of subpages to choose from) can not only overwhelm users living with cognitive problems, making it challenging to process and remember the content, but also impair decision-making skills by hindering the ability to effectively filter out the relevant information. This creates a poor user experience and can cause frustration and disorientation. In addition, it can compromise the usefulness and accessibility of such web pages because users may struggle to find the specific information they need within a cluttered interface, hindering their ability to use the website effectively. To increase usefulness and decrease information overload for people living with dementia, it is suggested to provide content that is comprehensive, practical, and reassuring [43]. Moreover, categories should be less broad and listed alphabetically [44]**.**

#### Scrolling

The need for scrolling caused participants to miss useful information and prevented successful task completion during the think-aloud sessions. A user with cognitive impairments can experience challenges in remembering the need to scroll every time a new web page is loaded. In addition, excessive scrolling can lead to increased cognitive load, as it requires processing information sequentially, potentially overwhelming the working memory. It has been suggested from observational data that reducing the cognitive load of a web page (eg, less information on a page) can contribute to tackling scrolling issues, as scrolling appears to have a high cognitive load [46]. Moreover, the physical need for scrolling may be challenging and cause fatigue [46].

#### Content and User Expectations

It was found that 48 usability problems related to user expectations were primarily caused by the discrepancy between user expectations and actual content organization. This led to frustration and confusion among participants, making it difficult to locate the information they sought. The findings emphasize the importance of accurately representing content and containing words that users feel comfortable with, especially in dementia-friendly DHTs. Future iterations of redesigning the website should focus on aligning user expectations with content presentation through co-design, which involves people living with dementia from the start of the process. This has been shown to be beneficial for both the person living with dementia and the design process itself [47-50]. However, a recent systematic review found that only 23% of studies involved people living with dementia in their design approach [51]. Additional recommendations to better align the content with the user expectations include considering a potential artificial intelligence–generated language check to ensure that all information relevant for patients is provided at the B1 level using: (1) a clear title and subheadings, (2) an active writing style with examples, (3) simple words that everyone knows, and (4) short and clear sentences. Moreover, terminology should be consistent, familiar, and comfortable and in Dutch (avoid English terms; for example “second opinion” was found to be confusing).

#### System Feedback

Incomprehensible or seemingly invisible system feedback can lead to confusion and difficulties with navigating a website. For example, this occurred when clicking on a main item on the home page or the menu that refreshes the current page without any visible changes. It hinders the ability to understand the system’s responses, making it challenging to navigate the website effectively. This lack of clarity also reduces users’ confidence in their interactions, potentially causing them to make errors. Providing visually clear, short, and positive instructions may improve the understanding of users living with MCI or dementia about the system feedback on their interactions with the website [9,43]. In essence, clear and meaningful system feedback is essential for providing guidance and minimizing user errors. To tackle these issues, it is recommended to clearly distinguish the clickable and nonclickable areas to prevent confusion or endless clicks. After pressing a clickable area, the consequences of this click should be clearly visible or audible. This distinction can be made with colors, icons, buttons, vibrations, or audible beeps.

### Limitations

This study had several limitations. First, the goal of the study was to conduct a heuristic evaluation with the DEMIGNED principles to investigate the reality of these principles in capturing usability problems that people living with dementia may experience. The realness was determined by mapping the findings from the think-aloud sessions to the findings from the heuristic evaluation. These insights suggest that 50% (18/36) of the findings by the experts were real usability problems experienced by potential end users. Even though this shows the potential of the DEMIGNED principles in heuristic evaluations, it may be possible that some usability issues identified from the heuristic evaluation were too subjective and hence not encountered in the user-testing method. This can be caused by methodological differences between the subjective nature of heuristic evaluations (as they predominantly rely on expert judgment founded on the DEMIGNED principles) and the objective user feedback derived from think-aloud tests involving direct user interactions. Second, an explanation as to why some usability problems from the heuristic evaluation were not encountered in the think-aloud sessions may be the fact that 18 tasks to guide the evaluators through most of the website were performed during the expert testing, while only 7 tasks were performed during user testing. Conducting 18 tasks was deemed to be too challenging in terms of cognitive overload, concentration, and motivation of the participants. The think-aloud tasks were composed with the aim of catching most of the pages that were evaluated during the heuristic evaluation. However, such a variety in the number of tasks could introduce bias in agreement scores calculations, such as Cohen κ, and was, therefore, left out of the analysis. Third, the software used for capturing the think-aloud sessions introduced some limitations. Due to the setup, participants were to use the smartphones provided by the researchers. This allowed the researchers to set up the screen-sharing function of the smartphone rather than going through the participants’ smartphone, which might cause stress or anxiety related to their privacy before starting the think-aloud session. This may introduce bias to the results, as the smartphone could be new for the participant. However, the study was conducted within an internet browser consistent across various smartphones. In addition, due to unannounced software updates, the sixth and seventh think-aloud session recordings suffered from audio issues. Therefore, only the observations from the video recordings were used in the analysis. However, saturation was reached, as no new usability issues were encountered during these sessions, which is important in think-aloud research [52]. Nevertheless, dementia does present a wide range of symptoms, so it might be possible that additional usability problems would arise for those with symptoms varying from the symptoms the participants experienced during the think-aloud sessions. Fourth, 1 user profile for the think-aloud session was created that captured a representative sample of people most likely to use the website of Alzheimer Center Amsterdam: those who visit the memory clinic to seek support for their cognitive complaints. The most frequent diagnosis for people aged <65 years, which is the focus of Alzheimer Center Amsterdam, is SCD, followed by the diagnoses of dementia and MCI [36]. This was reflected in the included sample for user testing. Therefore, we assume that with this group of participants, most usability issues were detected. However, these varying diagnoses may also influence the types and severities of usability problems. Nevertheless, in this study, the reality of the principles has been examined for this group as a whole, given the novelty of the DEMIGNED principles and their scientific basis of study, including people living with varying cognitive complaints, MCI, or dementia. Nevertheless, an approach to further investigate the reality of the DEMIGNED principles could be to compare the detected usability problems between people with varying levels of cognitive abilities and those without such cognitive issues to rule out the possibility that usability problems exist independently of cognitive abilities. Fifth, 1 evaluator only conducted approximately 50% of the tasks in the heuristic evaluation due to time constraints. This may have influenced the number of times a usability problem was encountered. However, the researcher was involved in the further analysis of the heuristic evaluation data. Finally, the sessions were conducted at the memory clinic. Even though we emphasized to the participants to imagine that they were in a home setting and allowed them to ask their partner or loved one for support, results may be different when this study is conducted in a home setting.

### Future Research

Future research should focus on further evaluating the use of the DEMIGNED principles in expert reviews to assess their effectiveness in identifying real usability issues experienced by people living with varying types and stages of dementia. For example, digital health tools for people living with dementia that offer different functionalities from those offered by the mobile information resource evaluated in this study should be evaluated using the DEMIGNED principles, such as health monitoring, medication management, or participating in leisure or social activities. This may support categorizing the applicability of DEMIGNED principles per digital health functionality, as not all principles apply to each digital health tool. For example, in this study, the principle “ensure no time pressure” was not violated, as it was not applicable for the website. In addition, it is important to further evaluate the relevance of DEMIGNED principles for other types of user interactions, such as input controls (text fields, dropdown lists, toggles, etc) and serious games. This hopefully leads to more accessible digital health tools and the validation and refinement of the DEMIGNED principles. In the short term, the recommendations for redesign should be implemented into a new prototype for the website. The prototype should again be evaluated to ensure its usability. To enrich this evaluation, eye tracking and emotion readers could be used in think-aloud sessions to obtain more insights into the user experience because verbalizing what they are doing can be challenging for people living with dementia [53]. The emotion readers could also support in further investigating potential stress reactions of these people while conducting tasks on the website. Moreover, more extensive user testing could be conducted with the redesigned website, including metrics such as task completion rates and times, to gain more insights into the usability of the website. These metrics have been suggested to produce the most reliable results when conducting user testing with people living with dementia [53]. Finally, the literature suggests that an early decline in cognitive function may be detected from the input that people deliver when using a search engine [54]. The findings from this study related to using the search functionality initiate future research opportunities to optimize the search engine’s functionality and explore opportunities to use this in research to detect cognitive decline.

### Conclusions

This study showed that applying the DEMIGNED principles in expert testing can capture usability problems that people living with SCD, MCI, or dementia can experience when using a mobile website. The think-aloud analysis revealed 10 additional usability issues that were not captured in the heuristic evaluation approach. This shows the importance of involving end users in usability evaluations. Moreover, these findings provided insights into refining the DEMIGNED principles, mainly related to the use of a search function and barriers caused by the user’s frame of mind. Future research should look into the applicability of the DEMIGNED principles for other functionalities that DHTs provide to increase the accessibility of digital health and decrease digital health inequity for this complex and rapidly increasing population.

## Appendix

Multimedia Appendix 1 Overview of unique usability issues captured from the heuristic evaluation.

## Abbreviations

DHT: digital health technology
MCI: mild cognitive impairment
MOLDEM-US: mobile health for older adults with dementia–usability framework
SCD: subjective cognitive decline
