# Supplementary material for: Capturing Usability Problems for People Living With Dementia by Applying the DEMIGNED Principles in Usability Evaluation Methods: Mixed Methods Study
Source: JMIR Hum Factors. 2024 Jul 31;11:e54032. doi: 10.2196/54032 (PMC11325128; doi:10.2196/54032)
Supplement: Multimedia Appendix 1 [file humanfactors_v11i1e54032_app1.docx]

**Multimedia Appendix 1**: Overview of unique usability issues captured from the heuristic evaluation

| **Specification** | | | **Problem** | Evaluators^a^ (n=5), n (%) | **Severity score^b^, average** | **Frequency of problem encounterd in TA^c^** | **Violated DP ^d,e^** |
| --- | --- | --- | --- | --- | --- | --- | --- |
| **Theme 1: cognition** | | | | | | | |
|  | ***Subtheme: navigation (n=11)*** | | | | | | |
|  |  | Search function results | The search function does not work properly as the user cannot find the desired page with a single search term. This can cause confusion and makes content unfindable. | 1 (20) | 2 | 12 | C-Navigation |
|  |  | Run search function | The search function does not run before *go* is pressed on the device’s keyboard. This causes the user’s inability to complete a task | 1 (20) | 4 | 3 | C-Navigation  C- Tutorials |
|  |  | User expectations | Information headings do not align with textual content. When searching for information, users may expect to find information under the wrong heading or page, causing confusion and difficulties in remembering where to find information | 5 (100) | 4 | 48 | C-Tutorials  C-Navigation  P-Elements  C-Monitoring  S-Understandability |
|  |  | Multiple structures | The “donate” and “team” pages have a different navigational structure from the other pages, which may cause frustration as the user cannot return to the previous page using the user-interface and an additional structure needs to be learned and remembered. | 2 (40) | 3 | 0 | C-Navigation  C-Monitoring |
|  |  | Linearity | There are several ways to reach a page rather than a linear structure. This causes confusion as the user will expect to receive new content with a new action rather than receiving the same content. | 1 (20) | 2 | 8 | C-Navigation |
|  |  | Duplicate information | Some information can be found on different pages. If someone recognizes the text, they can miss valuable information on the rest of the page and this may cause confusion in monitoring the user’s navigation | 2 (40) | 3 | 1 | C-Navigation |
|  |  | Scrolling | When the user clicks on a subject in the menu, they have to scroll down to see that the information has changed. This causes confusion, and the user might miss information. | 5 (100) | 4 | 9 | C-Navigation  P-Compartmentalize  P-System feedback |
|  |  | Menu structure | It is unclear to the user that the main page has subpages. The user might think that they are independent pages instead of related subpages, which can cause confusion. | 3 (60) | 2 | 0 | C-Navigation |
|  |  | Filter function on the team page | There is no logical order within the team page and the filter function does not work properly (eg, selecting all professions provides 6 people). There is no alphabetical order. Therefore, the team members are hard to find. | 3 (60) | 2 | 0 | C-Navigation |
|  |  | Patient portal  findability | A user is unable to find the patient portal on the homepage or in the menu and has to search and click on patient care to find the correct link. A user does not expect this and may therefore be unable to find the link to the patient portal. | 3 (60) | 2 | 0 | C-Navigation  C-Monitoring |
|  |  | External navigation | Some links on the website redirect the user to an external location, such as email, Google Drive (Google LLC), the patient portal, or YouTube (Google LLC). This may cause the user to lose the navigational structure. | 3 (60) | 4 | 2 | C-Navigation  C-Monitoring |
|  | **Subtheme: content presentation (n=5)** | | | | | | |
|  |  | Information overload | There is an overload of information on a single screen. This makes the page cluttered and information hard to find. | 4 (80) | 2 | 20 | C-Navigation  C-Adjustability |
|  |  | Menu items | Some headings have many subheadings, and the headings cannot be closed. This can cause an overload of information and overwhelm the user. | 3 (60) | 2 | 2 | P-Elements  P-Compartmentalize  P-Click ability  C-Navigation |
|  |  | Redundant webpages | Pages with limited information, mostly redirect to other pages. This may cause information overload. | 1 (20) | 2 | 0 | C-Navigation |
|  |  | Button use | Some buttons are implemented as an image or require the user to press a picture next to some text to read more. When buttons do not represent buttons, this can cause difficulties in interacting to complete actions. | 4 (80) | 2 | 0 | C-Icon use  P-Elements |
|  |  | Inconsistent information provision | The way information is provided on research projects and diagnostic tests is not consistent, which may lead to confusion, as it seems like the information is missing | 1 (20) | 2 | 0 | C-Monitoring |
|  | **Subtheme**: t**utorials** **(n=1)** | | | | | | |
|  |  | Support | There is no opportunity for the user to receive a tutorial on how to interact with the website. This may already cause the user to fail to complete any actions | 1 (20) | 4 | 0 | C-Tutorials  F-Support |
|  | **Subtheme: complexities (n=1)** | | | | | | |
|  |  | Plug-ins | External tools and plug-ins, such as the contact form can be cognitively challenging because of Captcha (“I'm not a robot”) or QR codes | 1 (20) | 4 | 0 | C-Tutorials  C-Abilities |
| **Theme 2: Perception** | | | | | | | |
|  | **Subtheme: interaction** **(n=2)** | | | | | | |
|  |  | Clickable areas | It is unclear whether parts of the website, such as tabs, menu items, or dropdown menus are clickable or unclickable. This causes confusion, making the user miss information | 4 (80) | 3 | 4 | C-Navigation  C-Icon use  P-Click ability  F-Positive feedback |
|  |  | System feedback | After the user presses certain items on a webpage, it is unclear whether the changes are made to the interface. This system feedback can go unnoticed, causing difficulties to continue. | 4 (80) | 3 | 19 | P-Click ability  C-Monitoring  C-Navigation  F- Positive feedback |
|  | **Subtheme: layout (n=4)** | | | | | | |
|  |  | Consistent interface | The layout is not consistent, for example the colors of headings. This can cause confusion. | 2 (40) | 2 | 0 | P-Compartmentalize |
|  |  | Font size | A user is unable to adjust the text size or automatically adjust to screen size settings of a device, making texts difficult to read. | 2 (40) | 3 | 3 | P-Elements |
|  |  | Colors | The “filter” function has a perceptive nonvisible color combination (blue and green) | 5 (100) | 2 | 1 | P-Color use  P-Elements |
|  |  | UI^f^ elements | Some UI elements overlay other buttons or are difficult to notice due to size, shape, or location making them difficult to read and interact with | 4 (80) | 3 | 0 | P-Elements |
|  | **Subtheme: embedded elements (n=3)** | | | | | | |
|  |  | Video | Some information videos are only half visible on the screen, such as the lumbar puncture and summer camp video. | 3 (60) | 3 | 0 | P-Compartmentalize  P-Elements |
|  |  | Text | Text is not always easily readable as they are not clearly visible on the screen. Moreover, due to evaluating the mobile version of the website, the text mentions incorrectly that information can be found on the left side of the page, which is above. Furthermore, some text surrounds images. This may cause difficulties reading. | 2 (40) | 3 | 0 | P-Compartmentalize |
|  |  | Pop-ups | Pop-ups cover a large part of the screen. When the user does not interact with a pop-up, the pop-up stays present and covers a large part of the screen, causing the user to miss interacting with the content | 2 (40) | 4 | 2 | P-Compartmentalize |
| **Theme 3: Frame of Mind** | | | | | | | |
|  | ***Subtheme: Content (n=4)*** | | | | | | |
|  |  | Directness | The subheadings about acceptance and information are now read as too commanding, but do not contain concrete action points. This may cause stressful reactions | 1 (20) | 3 | 0 | F-Content  S-Understandability |
|  |  | Confrontation | Video about living with Alzheimer's disease can be stressful (eg. euthanasia) when someone has complaints and is looking for information. | 2 (40) | 3 | 1 | F-Content |
|  |  | Empty content | Pages that lack information, are empty, or are erroneous can cause stress in further interactions. | 4 (80) | 0 | 3 | F-Content |
|  |  | Personalization | It is unclear which research projects are relevant for the user, which may lead to reading potentially irrelevant information. | 1 (20) | 1 | 1 | F-Preferences |
| **Theme 4: Speech and Language** | | | | | | | |
|  | ***Subtheme: Accessibility (n=4)*** | | | | | | |
|  |  | Text to speech | There is no option available for a user to read text out loud. Therefore, it is not inclusive. | 1 (20) | 2 | 0 | S-User input |
|  |  | Speech to text | Free-text inputs do not have the option for speech input | 2 (40) | 2 | 0 | S-User input |
|  |  | Language level | The jargon and technical terms used are not always in Dutch and are not on the B1 level. This makes it hard for people to read the text. | 4 (80) | 3 | 5 | S-Understandability |
|  |  | Abbreviations | Abbreviations, mainly those in research projects, are used before being explained | 1 (20) | 2 | 0 | S-Understandability |
|  | ***Subtheme: Consistency (n=1)*** | | | | | | |
|  |  | Language use | The language use is not consistent, for example multiple designations for “lumbar puncture” This can cause confusion. | 4 (80) | 2 | 0 | S-Understandability |

^a^ Number of evaluators who detected the usability problem. ^b^ Average severity score from evaluators based on Nielsen severity ranking [35]. ^c^ The number of times a usability problem was encountered by participants during the think-aloud sessions. ^d^ DP: design principle from DEMIGNED. ^e^ Each principle has an abbreviation presented with the first letter of the category, followed by a key word describing the principle. ^f^ UI = user interface:
